# Effect of Seat Backrest Inclination on the Muscular Pattern and Biomechanical Parameters of the Sit-to-Stand

**DOI:** 10.3389/fnhum.2021.678302

**Published:** 2021-09-09

**Authors:** Nadège Tebbache, Alain Hamaoui

**Affiliations:** Laboratoire CIAMS, Université Paris-Saclay, Saint Aubin, France

**Keywords:** sit-to-stand, backrest inclination, speed, ground reaction forces, electromyography, anticipatory postural adjustments

## Abstract

**Objectives:** The sit-to-stand (STS) transfer mobilizes an extended part of the kinematic chain throughout a postural phase characterized by a flexion of the trunk and a focal phase consisting of a whole-body extension. The aim of this study was to analyze the variations of the global muscular pattern and the biomechanical parameters in both phases, in relation with seat backrest inclination.

**Methods:** Fifteen participants were asked to stand up from a seat with 5 backrest inclination settings and at 2 execution speeds. The ground reaction forces and the activity levels of fifteen muscles of the trunk and lower limbs were investigated.

**Results:** Backrest-induced modifications were mainly observed in the postural phase: inclining the backrest backward increased the phase duration and the activity level of the sternocleidomastoideus and the rectus abdominis, while it reduced the activity of the tibialis anterior. It also allowed for an increased maximal anteroposterior velocity of the body center of mass. Higher execution speed led to increased and earlier muscular activities of many trunk and lower limbs muscles, predominantly in the postural phase.

**Discussion:** Taken together, these results suggest that a greater backrest inclination increases the demand in the postural phase due to the increase of the upper body gravity torque about the ischial tuberosities, and requires an adaptation of muscular activity levels and timing, but with the same overall pattern. The kinetic energy gained during the longer excursion of the trunk may also require less activation of the lower limbs muscles involved in the generation of propulsive forces of the body.

## Introduction

The sit-to-stand (STS) is the demanding and frequent transfer from the seated posture to the standing posture. From a kinematic point of view, the STS consists of a trunk flexion phase followed by an extension of the trunk and lower limbs initiated after seat unloading (Kelley et al., 1976; Nuzik et al., 1986; Rodosky et al., 1989; Hirschfeld et al., 1999; Boukadida et al., 2015). Integrating kinetics, Schenkman et al. (1990) assumed that trunk flexion moves the body center of mass forward but above all increases the upper body forward momentum. This momentum is then transferred into a whole-body vertical momentum once the seat is unloaded, allowing for whole-body extension.

The STS, a demanding task, requires the activation of a large number of muscles with appropriate coordination. Some common components of this muscular pattern can be extracted from electromyographic studies. The first muscle to be activated during the STS task is the tibialis anterior (TA) (Doorenbosch et al., 1994; Roebroeck et al., 1994; Vander Linden et al., 1994; Gross et al., 1998; Khemlani et al., 1999; Rodrigues-de-Paula-Goulart and Valls-Solé, 1999; Tebbache and Hamaoui, 2020). Its activity is associated with foot stabilization during trunk flexion (Doorenbosch et al., 1994; Roebroeck et al., 1994; Vander Linden et al., 1994; Gross et al., 1998; Khemlani et al., 1999). As in many others forward oriented tasks, it is also involved in the backward shift of the center of pressure in STS initiation, together with the inhibition of the soleus muscle (Sol) (Crenna and Frigo, 1991). Head (sternocleidomastoid) and trunk (abdominal muscles) flexors are recruited to perform the forward tilt of the trunk (Rodrigues-de-Paula-Goulart and Valls-Solé, 1999). Quadriceps, together with hamstrings, are then activated for seat unloading and lower limb extension, with head (upper trapezius) and spinal extensors guiding the verticalization of the whole body (Munton et al., 1984; Roebroeck et al., 1994; Vander Linden et al., 1994; Rodrigues-de-Paula-Goulart and Valls-Solé, 1999; Bouchouras et al., 2015; Chorin et al., 2016). Several studies reported that quadriceps, hamstrings and trunk extensors are the main driving forces of the sit-to-stand and are activated once the vertical projection of the center of mass has been brought closer to the feet or its speed is sufficient (Pai and Rogers, 1990; Vander Linden et al., 1994; Rodrigues-de-Paula-Goulart and Valls-Solé, 1999; Hof et al., 2005). Posterior lower leg muscles control the horizontal momentum and stabilize the posture at the end of the STS (Doorenbosch et al., 1994; Khemlani et al., 1999; Rodrigues-de-Paula-Goulart and Valls-Solé, 1999; Cuesta-Vargas and Gonzalez Sanchez, 2013). In addition, a recent study exploring the effect of backrest inclination on muscular activity showed an increase in the activity level of upper body flexors (abdominal muscles and SCOM) and ST, together with a decrease in TA activity before seat unloading (Tebbache and Hamaoui, 2020).

According to Gelfand revisiting Bernstein’s ideas (Bernstein, 1967), voluntary movements include a postural component related to stability and a focal component related to the voluntary movement itself. Postural activity happens during and after the focal movement, but mainly beforehand (Belenkii et al., 1967; Bouisset and Zattara, 1981; Cordo and Nashner, 1982) with Anticipatory Postural Adjustments (APAs). APAs precede the focal movement, and their assumed goals include compensation of the forthcoming perturbation associated with the focal movement (Bouisset and Zattara, 1987) as well as the generation of propulsive forces when the movement involves a change of support base (Herman et al., 1973; Breniere and Do, 1986; Brenière and Do, 1991; Stapley et al., 1998). For the STS task, APAs occurring during the postural phase are rather used for the latter purpose.

Two main phases can thus be distinguished during the STS task: one called postural phase, during which the trunk is flexed forward, which acts as a preparation for the other phase, when body extension takes place and seat unloading occurs, namely the focal phase (Diakhaté et al., 2013; Alamini-Rodrigues and Hamaoui, 2017; Hamaoui and Alamini-Rodrigues, 2017). In between those 2 phases is the seat-off, when seat unloading occurs.

It has been shown that APAs are motor-task specific and are organized according to a well-defined sequence. They adapt to initial conditions, execution conditions (including speed) and to the functional state of the system (Bouisset and Do, 2008). Therefore, the characteristics of the seat, which are a key factor in seated posture ergonomics, might induce APAs adaptations when performing the STS task. Several studies explored those determinants biomechanically, mainly seat height (Rodosky et al., 1989; Schenkman et al., 1996; Gillette and Stevermer, 2012; An et al., 2013; Yoshioka et al., 2014) and feet position (Shepherd and Koh, 1996; Khemlani et al., 1999; Kawagoe et al., 2000; Gillette and Stevermer, 2012; Ng et al., 2015). It was shown that lowering seat height and putting the feet forward resulted in greater joint constraints (Burdett et al., 1985; Rodosky et al., 1989; Arborelius et al., 1992). A lower seat led to increased trunk maximal angular velocity to increase upper body momentum generation (Hughes et al., 1994; Schenkman et al., 1996) or to failure in the case of elderly subjects unable to use the momentum transfer strategy (Hughes et al., 1994).

However, the influence of seat backrest inclination in terms of biomechanics remains to our knowledge understudied, although most resting and transportation seats have an inclined backrest. In this setting, trunk flexion is initiated against the force of gravity, instead of benefitting from it when the trunk is initially upright (Millington et al., 1992). It also extends the trajectory of the center of mass during STS, offering opportunity for the generation of a greater horizontal momentum in the postural phase, which is a fundamental component of the postural phase and of the STS strategy (Schenkman et al., 1990; Pai et al., 1994).

Consequently, the question arises as to how the motor pattern of the postural and focal phases of the STS is adjusted to seat backrest inclination. The first option might be a simple adaptation of muscular activity levels, while the other would involve an in-depth reorganization of the program with a new set of muscles being active at a different timing. Given the importance of momentum control during the STS task, and in order to analyze this task when maximal performance is sought, a special interest was also given to the execution speed parameter. It was expected that performing the STS at maximum speed would exacerbate and make more visible the adaptation of the motor program to backrest inclination.

Part of a similar question was addressed in a previous paper (Tebbache and Hamaoui, 2020), which gave a first indication of muscular variations in the postural phase. This paper aims to further deepen that analysis by an additional kinetics analysis of the STS task. The main hypothesis was that inclined backrest requires a higher level of muscular activity during the postural phase, but induces an increased horizontal momentum which may ease the focal phase.

## Materials and Methods

### Participants

Fifteen healthy subjects (7 males—8 females; age: 22.9 ± 3 years; weight: 65.8 ± 9.7 kg; height: 171.6 ± 7.4 cm, BMI: 22.2 ± 1.9 kg/m^2^), took part in this study. It was carried out in accordance with the recommendations of the local “Ethics Committee for Movement Analysis (CERAM), INU Champollion,” which has approved this study. All subjects gave written informed consent prior to the testing, in accordance with the Declaration of Helsinki. Due to a technical issue, one subject was then excluded from the results analysis.

### Experimental Set-Up

#### Customized Adjustable Seat

A specifically designed modular airline seat (part of a 2-seat row) was used in this study. It was made adjustable by ARTEC Aerospace company (Seilh, France) by modifying a regular airline seat to allow for the investigation of the influence of specific parameters. The backrest inclination, defined as the angle between the backrest and the seatpan, was adjustable in the range 90°–130° continuously.

#### Force Platform

**FIGURE 1:**
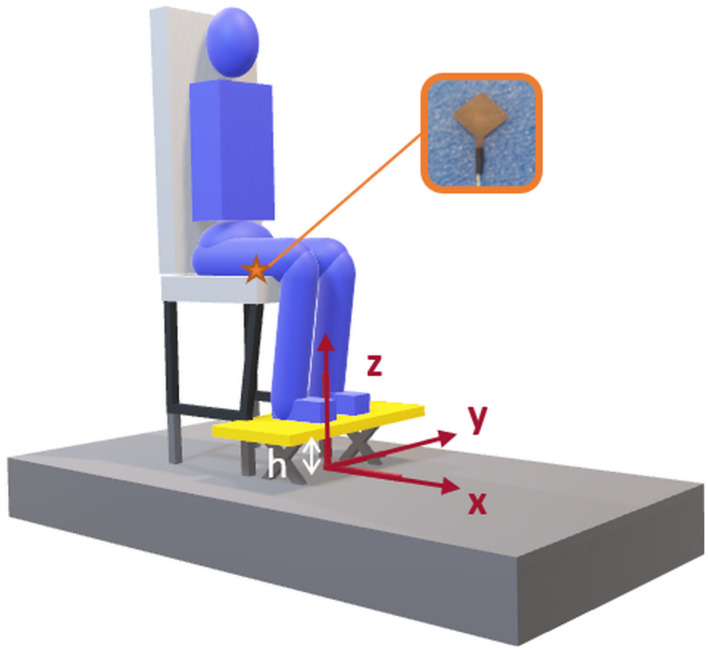
Seat on the force platform. Axes were as follows: *x*-axis is along the anteroposterior direction and points forward, *y*-axis along the mediolateral axis, pointing to the left, and *z*-axis follows the vertical direction, pointing upward.

A 1 m × 2 m 6-channel custom-made force platform (Bertec, Columbus, United States), on which the adjustable seat was screwed, was used to record forces and moments in the three orthogonal directions (Figure 1) with a sampling frequency of 1,000 Hz.

#### Pressure Sensor

A 25-mm^2^ capacitive pressure sensor (C500 sensor PPS, Los Angeles, United States) was inserted under the seatpan cushion, at mid-thigh level (Figure 1) and used to detect the onset of seat unloading.

#### Electromyography

A 16-channel wireless surface EMG device (Zero Wire Model, Aurion, Milan, Italy) was used. The signal was sampled at 1,000 Hz, amplified with a gain of 1,000, the bandwidth was 10–500 Hz, and the common mode rejection ratio 90 dB.

Surface EMG was collected on the dominant side of the subject (as told by the subject), on 12 muscles: Upper trapezius (TraS), sternocleidomastoideus (SCOM), neck extensors (NE), rectus abdominis (RA), erector spinae in the thoracic region (ES T6), erector spinae in the lumbar region (ES L3), rectus femoris (RF), vastus medialis (VM), semitendinosus (ST), gastrocnemius medialis (GM), tibialis anterior (TA), and soleus (Sol). Electromyograms were obtained using Ag/AgCl pre-gelled disposable electrodes positioned 2 cm apart over the muscle belly, in line with muscle fibers direction and on prepared skin. All electrode placements were confirmed using palpation and manual resistance tests, following SENIAM recommendations (Hermens et al., 2000).

Maximal voluntary contraction (MVC) electromyograms against manual resistance were recorded for amplitude normalization purposes, with two trials of 3 s for each muscle.

### Procedure

The experimental parameters were backrest inclination angle (90°–100°–110°–120°–130°) and execution speed [comfortable (CS)—maximal (MS)]. To standardize experimental conditions, the participants were barefoot and in their underwear. They first adopted a comfortable position on the seat, with their back resting against the backrest. Floor height was adjusted beforehand so that their thighs were horizontal, their feet flat on the floor, and their lower legs vertical. Feet placement was at a self-selected width, but the anterior-posterior position was imposed with the back of the heel at the rear end of the adjustable foot platform. Participants were asked to cross their arms loosely over their chest, then to stand up in response to a verbal signal, at the speed specified beforehand, namely comfortable or maximal. The comfortable speed was described as the natural self-selected speed used in daily life; the maximal speed was described as “as fast as possible.” They were instructed to perform an STS task shortly after a verbal signal was given, at a self-selected moment in time. Once in the standing posture, subjects had to keep still until an audio signal indicated the end of the trial. For each combination of the five backrest inclination levels and two execution speeds, five 6-s trials were performed, with a rest time of 10 s between trials and 120 s between series. Two training trials were implemented at each change of condition for the subjects to familiarize with the task of standing at the relevant speed and with a given backrest inclination.

Execution speed was randomized, but backrest inclination was performed in a systematic ascending order from 90° to 130°. Since the system of worm screw with crank used to set the backrest inclination at a precise level was very slow, randomization would have excessively lengthened the duration of the experiment.

### Data Analysis

#### Electromyography

EMG signals were full-wave rectified, filtered with a band-pass Butterworth filter (10–450 Hz) and smoothed (sliding window of 51 ms) (Conforto et al., 1999).

Muscle onset was detected using an algorithm based on the work of Lidierth (1986). The muscle was considered active when the mean amplitude of the EMG signal across the 50 following samples exceeded the baseline mean by 2 baseline standard deviations for more than 90 ms, without going below it for more than 15 ms. Baseline parameters were calculated on the 50 ms before the verbal signal was given. Movement start time, described below, was considered as the time origin and subtracted from the onset times obtained.

Activity levels were calculated as the average rectified values (ARV) of the EMG signal for each phase, normalized by the ARV of the 3-s MVC signal. Each muscle was thus characterized by a mean activity level for each phase, both expressed in percent of the MVC (A_*PP*_ and A_*FP*_).

#### Force Plate and Pressure Sensor Data

##### Center of Pressure (CoP)

The anteroposterior position of the center of pressure (CoP) was calculated from Equation 1, considering that the forces in the anteroposterior directions were applied at the height of the platform beneath the feet (h) (Figure 1), above the force platform. This simplification is addressed in the limitations section.

(1) $$x_{p}=\frac{hF_{x}-M_{y}}{F_{z}}$$

Considering

– *x*_*p*_ as the anteroposterior position of the CoP,
– *h* as the height of the adjustable platform beneath the feet,
– *F*_*x*_ as the total ground reaction force in the anteroposterior direction,
– *F*_*z*_ as the total ground reaction force in the vertical direction,
– *M*_*y*_ as the external moment along the mediolateral axis calculated at the center of the force platform.

##### Time Markers

Start time, end time, and seat-off time were measured to calculate the duration of the postural phase (dPP), the focal phase (dFP) and the entire STS (dSTS).

###### Start Time

**FIGURE 2:**
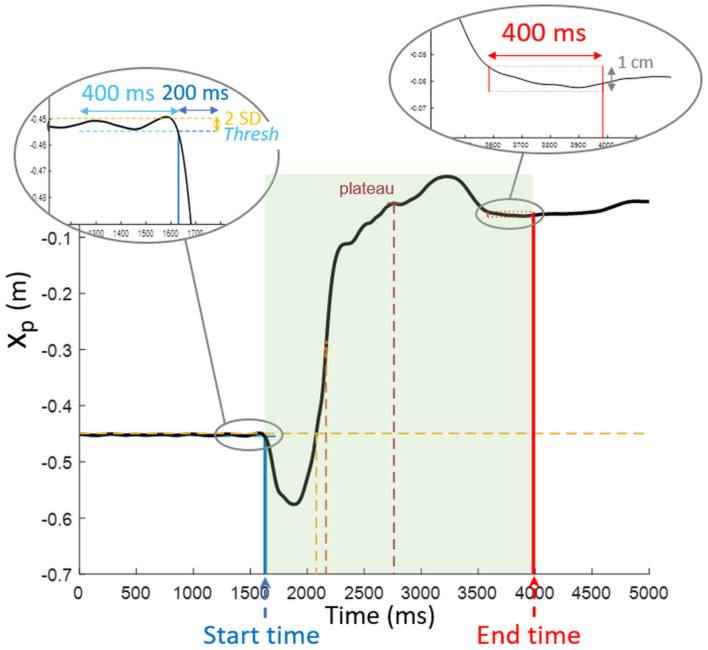
Detection of start and end times. Start time corresponded to the fall of x_*p*_ below the threshold calculated from the preceding 400 ms baseline for at least 200 ms. Concerning end time, the detection area was reduced to the time interval following the first plateau after feet loading, and end time preceded the first 400 ms where peak-to-peak CoP anteroposterior displacement did not exceed 1 cm.

The STS transfer start time was associated with CoP backward displacement initiation. It was the first time when all the values of a 200-ms window were lower than the mean value calculated in the preceding 400-ms sliding baseline window minus two standard deviations on this window (Figure 2). Start time was associated with the first value of the 200-ms window.

###### End Time

End time was detected based on CoP displacement as well. The algorithm included successive steps to determine the onset of the plateau following unloading, from which the CoP peak-to-peak amplitude on a 400 ms sliding window was calculated. When this value did not exceed 1 cm, the algorithm was stopped, and the end time was associated with the last sample of the window under consideration (Figure 2).

###### Seat-Off Time

**FIGURE 3:**
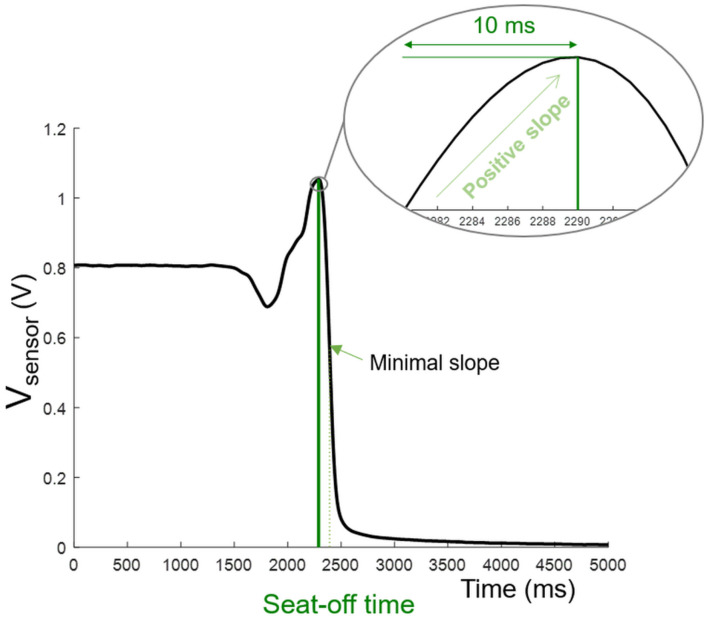
Seat-off detection. Starting from the time of occurrence of the steepest negative slope, the signal was analyzed backwards to identify the time when the slope changed sign and remained positive for at least 10 ms.

Seat-off time was detected by means of the pressure sensor inside the seatpan structure under the cushion. The algorithm detected the time associated with the steepest negative slope, and then moved back to detect the time when this slope changed sign and stayed positive for at least 10 ms, indicating the seat-off (Figure 3).

##### STS Indicators

**FIGURE 4:**
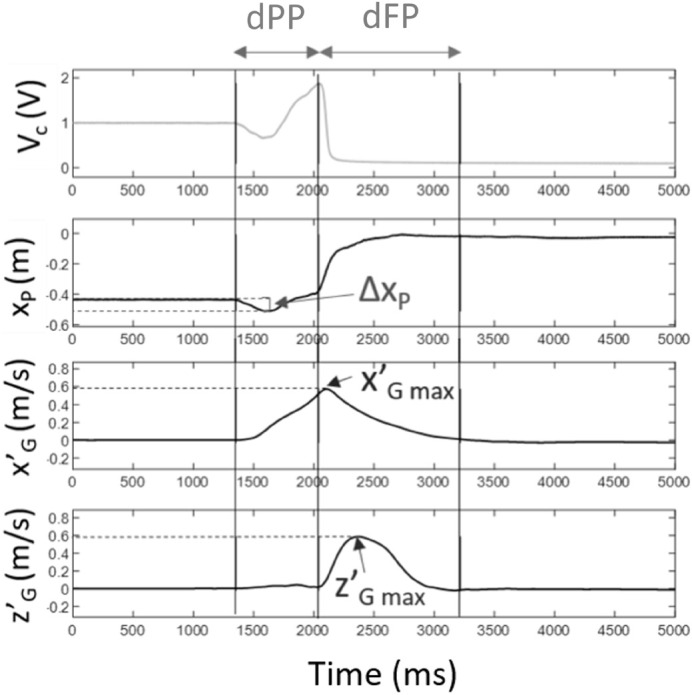
STS indicators. V_*sensor*_ represents the pressure sensor beneath the seat; x_*p*_ the position of the CoP, x′_*G*_ the anterioposterior velocity of the CoG; z’_*G*_ the CoG vertical velocity.

The STS was characterized by means of time, amplitude and velocity parameters:

– Anticipatory postural adjustments duration (dPP): time between start time and seat-off
– Focal phase duration (dFP): time between seat-off and end time
– Total STS duration (dSTS): time between start time and end time
– Anticipatory postural adjustments amplitude (Δx_*p*_): difference between the initial and the minimal anteroposterior position of the CoP during the postural phase.
– Maximal CoG speeds in the anteroposterior (x′_*G*_ max) and vertical (z’_*G*_ max) directions: peak of CoG speed signals obtained by integrating accelerations with null initial conditions (Figure 4).

Shorter times to perform the STS and higher maximal speeds were considered as indicators of a better performance, according to existing literature (Bouisset and Do, 2008; Diakhaté et al., 2013; Hamaoui and Alamini-Rodrigues, 2017).

#### Statistical Analysis

A 2-factor repeated-measures ANOVA was conducted for each dependent variable, with backrest inclination (5 modalities) and execution speed (2 modalities) as within-subjects factors, and the level of significance set at 0.05. When statistical significance was reached, simple contrasts were analyzed, by comparing each backrest inclination setting above 90° to the 90° setting.

## Results

Our results are presented in tables, completed by figures for the most relevant variations. The structure of the tables varies in order to highlight the significant results. When no significant interaction effect was found between the two independent variables, namely backrest inclination and velocity, a different table was made for each of these variables containing values averaged over the different modalities of the other variable.

### Electromyography

#### Onset Times

Most muscles investigated were firstly activated during the postural phase, with VM, RF, ST, and GM being activated close to the beginning of the focal phase. A significant effect of backrest inclination was observed on the onset times of 4 muscles, angles higher than 90° being associated with delayed onsets for VM (*p* < 0.001), and TA (*p* < 0.05), and TraS (*p* < 0.01 but with no significant contrast compared to 90°), and earlier onset of ES L3 (*p* < 0.01) (Table 1).

**TABLE 1:** Mean (SD) muscle onset times, in ms, for the 5 inclination conditions and for the 2 execution speeds, and *p*-values for effects of inclination (i), speed (V), and their interaction (i*V).

Muscle	V	90°	100°	110°	120°	130°	*p(i)*	*p(V)*	p(i*V)
NE	CS	358.9 (220)	335.1 (269.3)	310.2 (344.1)	321.8 (292.3)	355.6 (382.6)	NS	***	NS
	MS	114.6 (155.1)	76.8 (167.4)	118.5 (216.9)	111.9 (238)	65.5 (174.9)			
TraS	CS	168.9 (215.2)	123.7 (143.9)	140.6 (233.9)	126.3 (189.3)	163.1 (316.0)	**	NS	NS
	MS	9.4 (71.8)	26.3 (114.4)	15.7 (76.6)	18.9 (84.7)	9.1 (70.5)			
	***/90°***		***NS***	***NS***	***NS***	***NS***			
SCOM	CS	58.5 (443.8)	30.9 (379.3)	–46.7 (163.2)	–89.4 (39.4)	–121.1 (63.5)	NS	NS	NS
	MS	–45.8 (28.6)	55.9 (376.3)	–57.5 (25.8)	–66.7 (22.9)	–68 (30.2)			
RA	CS	105.1 (310.7)	–29.6 (76.8)	–60.7 (42)	–72.5 (55.3)	–17.2 (76.5)	NS	NS	NS
	MS	–16.3 (130.6)	–49.9 (32.1)	–53.7 (35.8)	–6.9 (119)	–34.9 (47.9)			
VM	CS	665.9 (205.3)	688.3 (163.5)	778.4 (201.4)	810.3 (185.7)	953.9 (257.9)	***	***	NS
	MS	293.2 (103.7)	315.8 (109.4)	332 (140.4)	398.2 (142.2)	477.3 (184.8)			
	***/90°***		***NS***	**	***	***			
RF	CS	569.8 (352.4)	520.1 (312.4)	590.1 (384.3)	462.8 (298.4)	610.8 (365)	NS	***	NS
	MS	225.3 (165)	197.1 (189)	206.6 (217)	190.7 (162.1)	256.9 (198.5)			
TA	CS	51.3 (100.7)	4.2 (48.4)	19.2 (55.1)	39.5 (29.3)	82.6 (127.3)	*	**	NS
	MS	26.8 (76.5)	–30.3 (12.3)	–27 (19.9)	1.2 (57.9)	0.7 (23.1)			
	***/90°***		**	*	***NS***	***NS***			
Sol	CS	480.8 (341.2)	443.3 (432.4)	576.6 (485.7)	523.7 (497.4)	667 (511)	NS	***	NS
	MS	153.8 (166.3)	172.3 (182.1)	163.1 (250.4)	122.7 (169.3)	199.3 (245.9)			
GM	CS	634.8 (331.9)	638.9 (268.3)	691.3 (349.5)	748.2 (474.8)	719.8 (472.9)	NS	***	NS
	MS	160.6 (101.6)	203.6 (160.7)	207.3 (196.8)	192.1 (218.5)	227 (234.4)			
ST	CS	655.9 (159.9)	726.5 (192)	822.7 (265.7)	787.3 (318)	893.6 (399.3)	*		**
	***/90°***		***NS***	*	***NS***	***			
	MS	272.1 (141)	302.3 (156.3)	340.5 (182.1)	323.8 (245.7)	280.1 (264.4)	NS		
ES T6	CS	280 (180.5)	273.9 (139.6)	273.9 (183.1)	267.4 (220.4)	253.6 (188.7)	NS	***	NS
	MS	150 (77.9)	124.9 (90.1)	112.8 (88)	100.7 (93.5)	105.6 (114.5)			
ES L3	CS	462.1 (182.6)	396.6 (111)	414 (225.4)	354.9 (145.1)	310.1 (142.7)	**	***	NS
	MS	148.9 (81.6)	131.5 (89.9)	113.3 (110.1)	93.7 (97.7)	81.5 (93)			
	***/90°***		***NS***	***NS***	**	***			

When the global effect of inclination is significant, the contrasts for each inclination compared to i0 is also shown (/90°). Symbols for the p-values are as follows: *p < 0.05; **p < 0.01; ***p < 0.001; NS: p ≥ 0.05.

When comparing the 2 speed conditions, performing at maximal speed significantly reduced the onset times of all muscles investigated except SCOM and RA.

Furthermore, the ANOVA revealed a significant interaction effect between backrest inclination × speed for ST (*p* < 0.01), with a significant simple main effect of backrest inclination only at comfortable speed (*p* < 0.05) but a significant effect of speed for each backrest inclination (*p* < = 0.01).

#### Activity Levels

When considering the mean activity levels, the most active muscles during the postural phase, showing values higher than 25%, were mainly located in the trunk (RA, ES L3, ES T6) and neck (NE, TraS) (Table 2). In the focal phase, these muscles were in contrast located in the lower limbs (VM, TA, Sol, GM), with decreased values for trunk and neck muscles (Table 3).

**TABLE 2:** Mean (SD) muscle activity levels in the postural phase (A_*PP*_), in % MVC, for the 5 inclination conditions, calculated for both execution speeds.

A_*PP*_	90°	100°	110°	120°	130°	*p(i)*
NE	35.03 (16.64)	36.97 (17.31)	37.86 (19.66)	39.06 (20.92)	38.13 (19.42)	NS
*p(/90°)*		–	–	–	–	
TraS	33.22 (23.75)	31.03 (22.39)	24.91 (13.77)	24.6 (14.15)	22.99 (13.61)	NS
*p(/90°)*		–	–	–	–	
SCOM	14.64 (13.53)	16.19 (13.75)	19.33 (14.3)	21.38 (14.16)	24.21 (13.89)	***
*p(/90°)*		NS	**	***	***	
RA	25.11 (20.86)	39.57 (37.31)	44.5 (29.94)	52.28 (38.53)	54.88 (36.49)	***
*p(/90°)*		**	***	***	***	
VM	3.84 (2.56)	3.86 (2.55)	3.96 (2.55)	3.95 (2.48)	4.82 (6.02)	NS
*p(/90°)*		–	–	–	–	
RF	5.45 (4.4)	6.07 (4.48)	5.97 (4.31)	6.04 (3.56)	5.45 (3.51)	NS
*p(/90°)*		–	–	–	–	
TA	22.31 (17.24)	21.09 (19.96)	20.15 (15.92)	19.25 (15.76)	16.9 (13)	**
*p(/90°)*		NS	NS	**	***	
Sol	19.04 (14.05)	16.41 (11.01)	19.01 18.17)	18.19 (12.49)	18.2 (12.69)	NS
*p(/90°)*		–	–	–	–	
GM	16.25 (19.44)	14.82 (16.95)	14.74 (16.09)	16.95 (16.02)	15.7 (15.61)	NS
*p(/90°)*		–	–	–	–	
ST	3.35 (2.59)	3.02 (2.1)	2.99 (1.89)	4.04 (3.05)	4.7 (4.82)	NS
*p(/90°)*		–	–	–	–	
ES T6	29.82 (43.78)	30.68 (51.41)	34.35 (60)	28.74 (34.18)	25.98 (30.47)	NS
*p(/90°)*		–	–	–	–	
ES L3	47.57 (57.32)	54.32 (56.65)	56.42 (62.69)	64.11 62.27)	69.26 (79.11)	NS
*p(/90°)*		–	–	–	–	

Symbols for the p-values are as follows: **p < 0.01; ***p < 0.001; NS: p ≥ 0.05. A dash (“–”) indicates that no statistically significant global effect was found, and therefore no further analysis was made.

**TABLE 3:** Mean (SD) muscle activity levels in the focal phase (A_*FP*_), in % MVC, for the 5 inclination conditions, calculated for both execution speeds.

A_*FP*_	90°	100°	110°	120°	130°	*p(i)*
NE	22.57	22.44	26.16	27.41	27.86	NS
	(12.45)	(11.84)	(19.37)	(22.17)	(21.72)	
*p(/90°)*		–	–	–	–	
TraS	10.42	9.62	9.08	8.98	9.55	NS
	(7.49)	(6.63)	(5.77)	(6.13)	(7.37)	
*p(/90°)*		–	–	–	–	
SCOM	3.25	3.18	3.78	3.52	3.55	NS
	(1.93)	(1.82)	(2.23)	(2.03)	(2.24)	
*p(/90°)*		–	–	–	–	
RA	5.61	5.82	5.63	5.73	5.85	NS
	(6.28)	(6.15)	(6.06)	(6.19)	(6.6)	
*p(/90°)*		–	–	–	–	
VM	33.15	30.62	31.49	30.10	30.34	NS
	(10.64)	(9.78)	(11.01)	(11.17)	(10.92)	
*p(/90°)*		–	–	–	–	
RF	16.51	15.04	15.98	15.45	15.97	*
	(8.06)	(7.47)	(8.24)	(7.51)	(8.28)	
*p(/90°)*		**	NS	*	NS	
TA	28.19	26.57	26.18	25.57	26.84	NS
	(21.63)	(20.19)	(17.88)	(18.92)	(22.01)	
*p(/90°)*		–	–	–	–	
Sol	34.72	32.4	31.35	30.22	32.85	*
	(16.25)	(14.93)	(15.48)	(13.88)	(18.26)	
*p(/90°)*		NS	*	**	NS	
GM	26.41	27.96	25.51	27.63	28.11	NS
	(22.46)	(26.65)	(23.60)	(23.94)	(24.48)	
*p(/90°)*		–	–	–	–	
ST	24.67	20.18	23.79	22.61	21.27	NS
	(18.13)	(12.32)	(18.44)	(18.00)	(14.87)	
*p(/90°)*		–	–	–	–	
ES T6	15.5	15.61	16.09	15.38	15.87	NS
	(9.28)	(9.01)	(9.40)	(7.95)	(8.70)	
*p(/90°)*		–	–	–	–	
ES L3	32.10	30.51	30.61	28.58	29.53	NS
	(11.45)	(10.26)	(12.18)	(8.18)	(8.81)	
*p(/90°)*		–	–	–	–	

Symbols for the p-values are as follows: *p < 0.05; **p < 0.01; NS: p ≥ 0.05. A dash (“–”) indicates that no statistically significant global effect was found, and therefore no further analysis was made.

**FIGURE 5:**
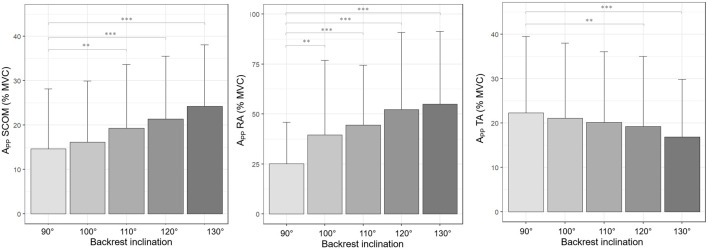
Mean (SD) muscle activity levels in the postural phase (APP), in % MVC, for the 5 inclination conditions calculated for both execution speeds. Symbols for the *p*-values are as follows: ^∗∗^*p* < 0.01; ^∗∗∗^*p* < 0.001.

Increasing backrest inclination resulted in significant variations of activity levels during the postural phase, with higher values for SCOM (*p* < 0.001) and RA (*p* < 0.001), and lower for TA (*p* < 0.05) (Table 2 and Figure 5). For this muscle, a significant decrease in activity was observed only from 120° on.

In the focal phase, increasing backrest inclination only led to lower activity levels for RF (*p* < 0.05) and Sol (*p* < 0.05) (Table 3).

Execution speed significantly increased activity levels in the postural phase of all muscles investigated except for TA and ES T6, although their mean activity level still increased in condition MS (Table 4).

**TABLE 4:** Mean (SD) muscle activity levels in the postural phase (A_*PP*_) and in the focal phase (A_*FP*_) in % MVC for the 2 speed conditions: comfortable speed (CS) and maximal speed (MS), calculated for the 5 backrest inclination levels.

	A_*PP*_			A_*FP*_		
	CS	MS	*p(V)*	CS	MS	*p(V)*
NE	27.20	47.62	***	24.91	25.66	–
	(14.00)	(16.99)		(15.75)	(19.99)	
TraS	19.62	35.08	***	10.01	9.05	–
	(10.39)	(21.07)		(6.72)	(6.54)	
SCOM	10.24	28.06	***	3.11	3.8	–
	(6.49)	(14.10)		(1.66)	(2.32)	
RA	27.11	59.43	**	5.29	6.16	*
	(18.85)	(38.58)		(6.19)	(6.1)	
VM	3.31	4.86	*	28.49	33.79	***
	(3.94)	(2.79)		(9.73)	(10.88)	
RF	4.28	7.31	***	14.16	17.42	***
	(2.9)	(4.42)		(7.33)	(8.01)	
TA	18.65	21.23	–	25.71	27.63	–
	(15.08)	(16.35)		(20.98)	(18.89)	
Sol	11.67	24.67	**	28.99	35.63	*
	(6.7)	(15.79)		(12.96)	(17.46)	
GM	8.25	23.14	***	20.39	33.87	**
	(7.91)	(19.59)		(17.45)	(27.52)	
ST	2.66	4.59	**	17.35	27.65	**
	(1.76)	(3.8)		(10.12)	(19.59)	
ES T6	17.5	42.32	–	16.27	15.11	–
	(16.24)	(58.75)		(9.5)	(7.97)	
ES L3	33.36	83.31	***	28.98	31.55	–
	(34.07)	(75.74)		(7.75)	(12.1)	

Symbols for the p-values are as follows: *p < 0.05; **p < 0.01; ***p < 0.001; NS: p ≥ 0.05. A dash (“–”) indicates that no statistically significant global effect was found, and therefore no further analysis was made.

During the focal phase, only half of the muscles investigated showed any significant variation according to execution speed, with a systematically higher activity in MS condition: RA (*p* < 0.05), RF (*p* < 0.001), VM (*p* < 0.001), Sol (*p* < 0.05), GM (*p* < 0.01), and ST (*p* < 0.01) (Table 4).

No statistically significant effect of the interaction between execution speed and backrest inclination was found.

### STS Indicators

#### Durations

The total duration of the STS increased with backrest inclination (*p* < 0.01) and decreased with execution speed (*p* < 0.001) (Table 5), but some variations were also specific to each phase of the STS.

**TABLE 5:** Mean (SD) total durations of the STS (dSTS), duration of the postural phase (dPP), maximal CoG anteroposterior velocity (x′_*G max*_), for the 5 inclination conditions and for the 2 speed conditions: comfortable speed (CS) and maximal speed (MS).

	dSTS (ms)	dPP (ms)		dFP (ms)	x′_*G max*_ (mm/s)	z’_*G max*_ (mm/s)
		CS	MS			
**90°**	2,267 (436)	738 (154)	374 (71)	1,711 (276)	578 (118)	706 (217)
**100°**	2,393 (379)	775 (149)	407 (97)	1,802 (316)	601 (111)	693 (194)
**110°**	2,342 (477)	851 (175)	463 (98)	1,685 (292)	612 (112)	706 (186)
**120°**	2,487 (430)	939 (150)	525 (110)	1,755 (304)	626 (108)	721 (196)
**130°**	2,553 (476)	1,080 (163)	610 (124)	1,707 (313)	630 (14)	709 (190)
*p(i)*	**	***	***	NS	***	NS
*p(100°/90°)*	NS	NS	*	–	*	–
*p(110°/100°)*	NS	***	***	–	***	–
*p(120°/110°)*	**	***	***	–	***	–
*p(130°/120°)*	***	***	***	–	***	–
*p(V)*	***	***		**	***	***
p(i*V)	NS	**		NS	NS	NS

For dPP, speed conditions were considered separately as the interaction effect i x V was significant. Symbols for the p-values are as follows: *p < 0.05; **p < 0.01; ***p < 0.001; NS: p ≥ 0.05. A dash (“–”) indicates that no statistically significant global effect was found, and therefore no further analysis was made.

When considering the focal phase, its duration was shortened at maximum speed (*p* < 0.01), with no effect resulting from backrest inclination. When focusing on the postural phase, an interaction was found between the 2 factors (inclination × speed) (*p* < 0.01), requiring a specific analysis of the simple main effect of each variable for each different setting. Backrest inclination significantly lengthened postural phase duration under both speed conditions (*p* < 0.001), and maximal speed produced the reverse effect for all inclination levels (*p* < 0.001) (Table 5).

#### CoP Maximal Backward Displacement (Δx_*P*_)

**FIGURE 6:**
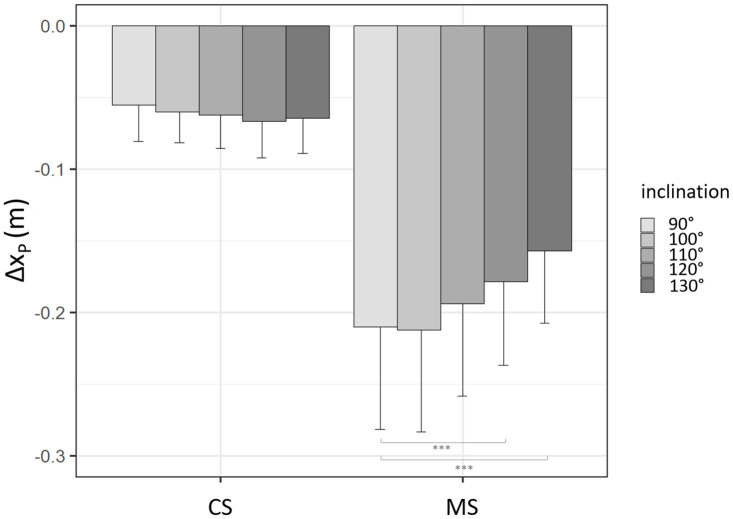
Maximal CoP backward displacement as a function of speed and backrest inclination. Mean and standard deviations are presented for the 2 speed conditions and the 5 backrest inclination levels. ^∗∗∗^*p* < 0.001.

An interaction effect inclination × speed was evidenced by the ANOVA (*p* < 0.001) (Figure 6), requiring that each simple main effect be analyzed separately.

Backrest inclination had a significant effect only in the condition MS (*p* < 0.001), with CoP maximal backward displacement decreasing with backrest inclination at 120° inclination and 130° inclination compared to the 90° level (Figure 6 and Table 6).

**TABLE 6:** Δx_*P*_ values for the 5 inclination conditions and for in the 2 speed conditions: comfortable speed (CS) and maximal speed (MS).

	Δx_*P*_ (mm)	
	CS	MS
**90°**	55 (25)	210 (72)
**100°**	60 (22)	212 (71)
**110°**	62 (23)	194 (65)
**120°**	67 (26)	178 (58)
**130°**	65 (24)	157 (50)
***p(i)***	**NS**	***
*p(100°/90°)*	–	NS
*p(110°/100°)*	–	NS
*p(120°/110°)*	–	***
*p(130°/120°)*	–	***
***p(V)***	***	
p(i*V)	***	

Symbols for the p-values are as follows: ***p < 0.001; NS: p ≥ 0.05. A dash (“–”) indicates that no statistically significant global effect was found, and therefore no further analysis was made.

**FIGURE 7:**
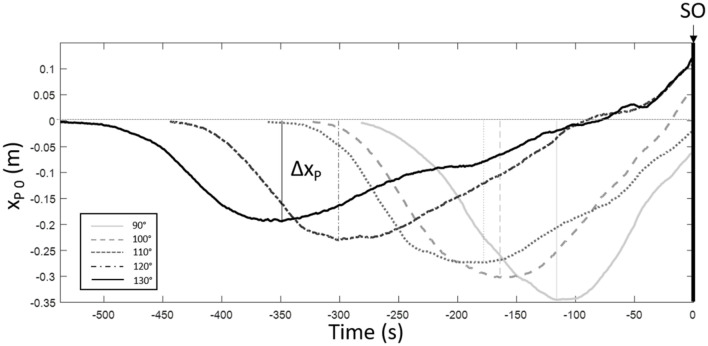
Displacement of x_*p*_ during the postural phase (until SO, seat-off time) as a function of time for one subject for the 5 backrest inclinations.

The effects of backrest inclination on CoP parameters could be observed in the raw data presented in Figure 7, with smaller and earlier excursion of CoP traces associated with increased inclination.

Execution speed had a significant effect at each inclination level with greater CoP maximal backward displacement at MS (*p* < 0.001) (Table 6).

Δx_*P*_ decrease with backrest inclination at maximal speed can be observed in Table 6 and from the raw data in Figure 7.

#### CoG Maximal Forward Velocity

Maximal CoG forward velocity (x′_*G max*_), reached around seat-off, significantly increased with backrest inclination (*p* < 0.001) (Table 5) and with execution speed (*p* < 0.001). The contrast analysis showed that all backrest inclination levels higher than 90° caused significantly higher x′_*G max*_ compared to the 90° level.

#### CoG Maximal Vertical Velocity

CoG maximal vertical velocity (z’_*G max*_), which was reached during the focal phase, was affected by speed conditions (*p* < 0.001), with larger values at MS (853 ± 159 mm/s at MS vs. 561 ± 85 mm/s at CS) (Table 5). However, no variation was observed according to backrest inclination.

## Discussion

### Effects of Backrest Inclination

Results calculated from EMG and force plate signals revealed that the increase in backrest inclination levels caused significant variations of both muscular activity and biomechanical parameters during the STS.

With a reclined backrest, the duration of the postural phase increased. This phenomenon can be related to the increased range of motion of the trunk when it is initially more extended, under the hypothesis of a limited variation of movement velocity. Consistently, onset times were also delayed with backrest inclination for VM and ST, which are part of the prime movers of body extension (Roebroeck et al., 1994; Vander Linden et al., 1994; Rodrigues-de-Paula-Goulart and Valls-Solé, 1999).

In accordance with a recent study (Tebbache and Hamaoui, 2020), differences in mean muscular activity levels were mainly observed during the postural phase, with higher values for two trunk (RA) and neck (SCOM) muscles when the trunk was more inclined backward.

These variations might be explained by a larger initial torque of the force of gravity about the ischial tuberosities, as the lever arm of upper body weight is larger when the trunk is further extended, and therefore a higher level of muscular activity is required to perform trunk flexion. Moreover, the onset time of ES L3 was shorter when the backrest was more inclined, suggesting earlier involvement of the lower back muscles in order to generate forces at the lumbar level to counteract gravity.

This increased muscular demand during the postural phase led to a larger x′_*G max*_, which took place shortly after the seat-off. Smaller APAs amplitude and TA activity level could be related to this higher momentum gained during the extended course of trunk flexion.

More specifically, these results suggest that with increased trunk range of motion and velocity, the role played by TA is taken over by trunk muscles (specifically flexors, SCOM and RA) and becomes less essential for STS success.

Indeed, as observed in our previous study (Tebbache and Hamaoui, 2020), TA activity level was reduced with backrest inclination in the postural phase, with no variation during the focal phase. Its activation was also delayed when the backrest was inclined, suggesting a lower participation in the STS task, as observed by various authors when the initial geometrical configuration deviated from the standard one (Doorenbosch et al., 1994; Vander Linden et al., 1994; Khemlani et al., 1999; Rodrigues-de-Paula-Goulart and Valls-Solé, 1999). Indeed, TA is involved in the backward shift of the center of pressure at STS initiation, occurring together with the inhibition of the soleus muscle (Sol) (Crenna and Frigo, 1991). This functional unit thus results in a larger lever arm of the ground reaction force about the ankle joint, creating an angular external moment which facilitates the propulsion of the body by breaking the rotation equilibrium in whole-body movements such as the STS task, gait initiation or pushing ramp efforts (Breniere and Do, 1986; Crenna and Frigo, 1991). The analysis of CoP trajectory showed that the initial backward shift (Δx_*P*_), which is considered as an indicator of APAs amplitude (Bouisset and Do, 2008; Diakhaté et al., 2013; Hamaoui and Alamini-Rodrigues, 2017), had smaller values when the backrest was more inclined, but only in MS condition.

This tends to further confirm the reduced necessity to generate those APAs, because they would have been less efficient given the initial segmental configuration and would even be counterproductive for fast hip flexion.

The compensation by the increased horizontal momentum should be enhanced at maximal speed, as the momentum gained is larger.

Furthermore, these results can be analyzed in light of the three mechanisms by which balance of a standing human can be maintained (Hof, 2007): displacing the CoP with respect to the vertical projection of the CoG (first mechanism), rotating a body segment with respect to the CoG (second mechanism) and applying an external force other than the ground reaction force (third mechanism) (Hof, 2007). Those three mechanisms contribute to modifying CoG acceleration. In the present study, it was observed that counter-rotating the trunk around the CoG (thus using the second mechanism) reduced the need for larger displacement of the CoP within the base of support (first mechanism).

The adjustments taking place during the postural phase and leading to a larger horizontal velocity allow for the maintenance of a globally unaltered focal phase, as suggested by its unchanged duration and CoG maximal vertical velocity.

Indeed, in contrast with x′_*G max*_, z’_*G max*_, which was reached later during the focal phase, was unaffected by backrest inclination, probably because the main effect of trunk flexion occurred along the anterior-posterior axis. Although the momentum transfer strategy suggests a transfer of the horizontal momentum to the vertical momentum (Schenkman et al., 1990), it seemed not to be entirely so in this study.

These results rather suggest a separate programming of the two phases although they are biomechanically interdependent.

It must be noted that the increase of postural phase duration in conditions of more inclined backrest does not necessarily represents a reorganization of the postural adjustments, as the course of trunk flexion is larger, and then automatically longer for a same execution speed.

### Effects of Execution Speed

The speed factor was initially selected to highlight the effects of backrest inclination, but speed effects were also studied thoroughly. Results showed that all muscles, except TA and SCOM, which were the first to be activated, presented a reduced onset time at maximal speed. Higher levels were observed for 10 muscles out of 12 during the postural phase, and for 6 out of 12 during the focal phase, suggesting a predominant effect in the former, as observed in previous work (Tebbache and Hamaoui, 2020). These variations in the muscular pattern resulted in a shorter duration of both postural and focal phases, with an increase of Δx_*P*_, x′_*G max*_ and z’_*G max*_ values. This way, earlier and higher muscular activity allowed for larger anticipatory postural adjustments and a better performance along the anterior-posterior and vertical axes. In contrast with the existing literature, which has depicted a more pronounced variation of vertical linear momenta compared to horizontal momenta (Pai and Rogers, 1988, 1990; Pai et al., 1994; Gross et al., 1998), our data showed the same levels of variation between CoP velocity peaks. Hence, the theory according to which horizontal momentum is limited due to balance constraints requiring the subject to stand straight at the end of the movement (Pai and Rogers, 1990) cannot be extended to conditions where the trunk was initially inclined backward.

### Adaptability of STS Parameters

Taken together, results from EMG and force plate data showed an adaptation of the STS strategy driven by the new biomechanical demand related to backrest inclination and execution speed.

A more extended initial position of the trunk was associated with increased muscular activity which led to increased kinetic energy gained through a wider range of trunk flexion, lowering the need of APAs.

A similar analysis could be made in respect of execution speed, showing an increased activity of all recorded muscles during the postural phase, and an increase in APAs magnitude and CoG velocity peaks.

These adaptations to biomechanical factors, rather than an in-depth reprogramming of the task, are in line with existing literature, which showed that an increase in ischiofemoral contact area with the seat (Diakhaté et al., 2013), a reduction in cervical (Hamaoui and Alamini-Rodrigues, 2017) or lumbar mobility (Alamini-Rodrigues and Hamaoui, 2017) led to modifications in APAs amplitude or duration and to lower motor performance. An insight of this result was observed in our previous study (Tebbache and Hamaoui, 2020), with muscular variations concordant with the initial geometrical configuration and mainly confined to the muscles active in the postural phase.

However, it must be noted that the increase of postural phase duration in conditions of more inclined backrest does not necessarily represents a reorganization of the postural adjustments, as the course of trunk flexion is larger, which naturally extends the duration of the postural phase for a same execution velocity.

From a more conceptual point of view, our findings also support the key principles that postural adjustments are task-specific, adaptable and under the control of the central nervous system (Bouisset and Do, 2008). Such ability requires an internal representation of the biomechanical parameters of the human body and their integration in motor programming.

### Practical Implications

Reclining the backrest, which increases static comfort due to a better distribution of the pressure across the soft tissue in contact with the seat (Hertzberg, 1972; Andersson et al., 1975; Barbenel, 1992) and to a decreased component of the gravity force on the spine (Andersson et al., 1979), has implications regarding the completion of the STS. It concerns the postural phase, with a need for higher activity of neck and trunk flexors (SCOM and RA) that could be a limiting factor for older people or patients presenting a weakness in these muscles. As a consequence, one can question the interest of bringing the backrest at 90° before performing the task, especially for people suffering a limited function of trunk and neck muscles. However, setting backrest inclination must then be easy to perform and much secure (slow velocity, no accidental triggering…), which is a challenging objective.

When considering the increase of kinetic energy for higher ranges of trunk flexion, which are associated with a lower need of TA and RF activity, it might in theory be beneficial to patients suffering lower limbs disorders, but only if trunk and neck muscles function is preserved.

## Limitations

The main limitation of the study design was the absence of kinematical data, which did not allow for a segmental analysis of osteo-articular mobility and its association with the muscular pattern. The sample size was also relatively small, but the population was homogeneous in terms of age and BMI. Regarding data analysis, the method was oversimplified and approximations were made in calculating the position of the CoP and the CoG, but the consequences can be considered as negligible because the system was at postural equilibrium prior to movement initiation.

Another limitation might be the order of the tasks which was partly randomized (execution speed but not the backrest inclination angle) due to the technical constraints of the customed seat. However, the 120 s rest time between series and the repeated stimulation of the subject all along the experiment should have minimized this side effect.

It must also be noted that the postural phase is longer and probably more conscious in the STS than in many other tasks exploring the postural adjustments (rising on tip toes, pointing, gait initiation…). As a consequence, one can question the possibility that the variations of the postural phase observed in maximum velocity condition might rather represent the compliance to the experiment instructions than a postural adjustment supporting the focal phase. However, the participants were asked to reach the standing posture as fast as possible, and not to perform the two phases quicker. With the focus on the goal, they were given the freedom to adapt postural phase parameters to the focal phase.

## Conclusion

This study revealed that the variations in STS programming associated with inclined backrest mainly consist in a simple and direct adaptation to the new biomechanical demand, with no in-depth reprogramming. It mainly raised the activity level of trunk and neck flexors due to the augmented torque of gravity about the ischial tuberosities center, but it also lowered the recruitment of some lower limbs muscles (TA, RF) thanks to the increased kinetic energy gained during the longer trunk flexion. Hence reclining the backrest should make the task more demanding at trunk level but less demanding for the lower limbs.

## Data Availability Statement

The original contributions presented in the study are included in the article/Supplementary Material, further inquiries can be directed to the corresponding author/s.

## Ethics Statement

The studies involving human participants were reviewed and approved by the Ethics Committee for Movement Analysis (CERAM), INU Champollion. The patients/participants provided their written informed consent to participate in this study.

## Author Contributions

AH wrote the initial research project. NT and AH contributed to conception and design of the study. NT organized the database. Both authors contributed to manuscript revision, read, and approved the submitted version.

## Conflict of Interest

The authors declare that the research was conducted in the absence of any commercial or financial relationships that could be construed as a potential conflict of interest.

## Publisher’s Note

All claims expressed in this article are solely those of the authors and do not necessarily represent those of their affiliated organizations, or those of the publisher, the editors and the reviewers. Any product that may be evaluated in this article, or claim that may be made by its manufacturer, is not guaranteed or endorsed by the publisher.
